# The stabilization of visibility for sequentially presented, low-contrast objects: Experiments and neural field model

**DOI:** 10.1167/jov.23.8.12

**Published:** 2023-08-16

**Authors:** Howard S. Hock, Gregor Schöner

**Affiliations:** 1 Department of Psychology and the Center for Complex Systems and Brain Sciences, Florida Atlantic University, Boca Raton, FL, USA; 2 Institute for Neural Computation, Ruhr-Universität Bochum, Bochum, Germany

**Keywords:** stabilization, visibility, bistability, neural dynamics, sequential effects

## Abstract

In any environment, events transpire in temporal sequences. The general principle governing such sequences is that each instance of the event is influenced by its predecessors. It is shown here that this principle is true for a fundamental aspect of visual perception: visibility. A series of nine psychophysical experiments and associated neural dynamic simulations provide evidence that two non-stimulus factors, self-excitation and short-term memory, stabilize the visibility of a simple low-contrast object (a line segment) as it moves over a sequence of unpredictable locations. Stabilization was indicated by the very low probability of visible-to-invisible switches, and dependence on preceding visibility states was indicated by hysteresis as the contrast of the object was gradually decreased or increased. The contribution of self-excitation to stabilization was indicated by increased visible-to-invisible switching (decreased hysteresis) following adaptation of the visibility state, and the contribution of memory to stabilization was indicated by visibility “bridging” long blank intervals separating each relocation of the object. Because of the unpredictability of the relocations of the object, its visibility at one location pre-shapes visibility at its next location via persisting subthreshold activation of detectors surrounding the low-contrast object. All effects were modeled, including contributions from adaptation and recurrent inhibition, with a single set of parameter values.

## Introduction

**Figure 1.**
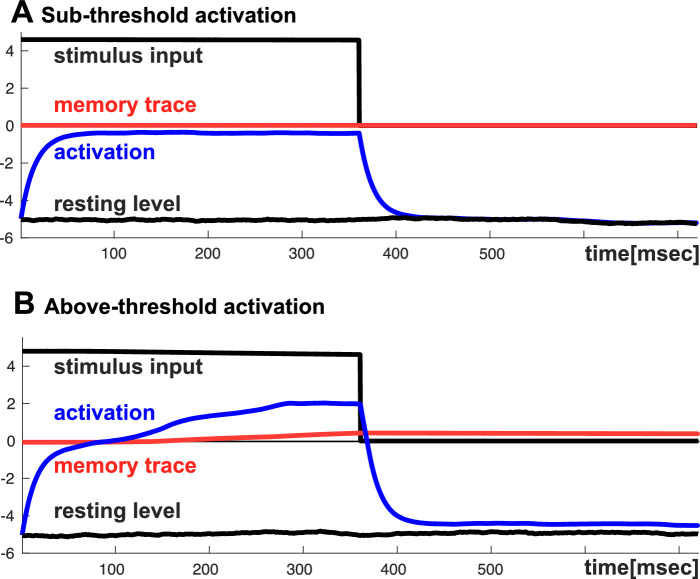
Time courses of detector activation (blue) in response to a stimulus presented for 360 ms (black), followed by a blank interval of the same length. (**A**) The stimulus is not sufficient to bring activation from the resting level (black line at the bottom) to levels above the threshold (at zero). No memory trace (red) is formed, and activation returns to the fluctuating resting level after the stimulus is removed. (**B**) The slightly stronger stimulus brings activation above threshold, engaging self-excitation (more activation than accounted for by adding stimulus input to the resting level). A memory trace arises, which prevents activation from fully returning to the fluctuating resting level after the stimulus is removed. (The time courses originate from the model presented in the General Discussion section.)

Maintaining the visibility of near-threshold, low-contrast objects is an ongoing challenge to the visual system. Small differences in background-relative luminance contrast can determine whether an object is visible or not, and random fluctuations in detector activation potentially result in the visibility of low-contrast objects hovering around the visibility threshold, stochastically switching back and forth between visibility and invisibility. Further challenges to the stabilization of visibility come from sequential changes in the retinal location of low-contrast objects (for example, as a result of a series of micro-saccades) (e.g., Ratliff & Riggs, 1950), momentary interruptions in stimulation (for example, as a result of the object's temporary occlusion by another object), and the suppressive effect of adaptation on visibility (e.g., Hammett, Snowden, & Smith, 1994). In order for stabilization to be fully realized, the visibility of a low-contrast object must carry-forward in time despite random fluctuations in detector activation, random changes in retinal location, and interruptions in stimulation. Perception at near-threshold contrast levels would be severely impaired were it not for processes that stabilize and maintain visibility over time and space. The current study has identified two such processes:

*Self-excitation*—When stimulus-initiated activation approaches the visibility threshold, visibility is stabilized by excitatory interactions among activated detectors. Such self-excitation boosts activation sufficiently above the visibility threshold to minimize the de-stabilizing effect of random fluctuations. Activation remains below the visibility threshold when random fluctuations prevent stimulus-initiated activation from reaching levels at which excitatory interactions are elicited. The evolution of these alternative activation states is illustrated in the two panels of Figure 1.

*Short-term memor*y—Low-pass temporal filtering of detector activation lays down a memory trace that increases detector activation after an object has been presented. This subthreshold memory trace increases the likelihood that visibility will be restored when the object reappears. As illustrated in Figure 1B, detector activation would quickly return to the no-stimulus resting level if it were not for the memory trace. These processes, which stabilize suprathreshold detector activation (and thus visibility), are potentially opposed by processes that suppress detector activation: recurrent inhibition and adaptation. These also were examined.

The test objects in the experiments reported in this article were line segments, stimuli well known to activate ensembles of cortical neurons in area V1 with similar orientation selectivity (Hubel & Weisel, 1962; Chapman, Zahs, & Stryker, 1991; Deneve, Latham, & Pouget, 1999; Mooser, Bosking, & Fitzpatrick, 2004). For these stimuli, self-excitation could take the form of excitatory interactions within such detector ensembles. Accordingly, mutual excitatory interactions would amplify stimulus-initiated activation within the ensemble, with the “extra” activation boosting the most strongly activated ensemble members above the visibility threshold.

The experiments in Part 1 of this study provided evidence for the excitatory interactions that are the basis for activation-boosting self-excitation, as well as short-term memory, the basis for the persistence of subthreshold activation. In Part 2, it is shown that low-contrast visibility is indeed stabilized by both self-excitation and the formation of memory traces when low-contrast line segments are presented over a sequence of randomly determined locations. These experiments show that the visibility of a stimulus is not determined independently at each of its locations but instead depends on processes that had affected visibility at its preceding location. The experiments in Part 3 investigated how this linkage between successive presentations is established. Finally, the General Discussion in Part 4 includes simulations of a dynamic neural field model that accounts for all of the experimental results with the same set of parameter values. The model incorporates self-excitation, short-term memory, inhibition, and adaptation.

## General methods

Stimuli were presented on an NEC MultiSync FB2141^SB^ 75-Hz monitor (NEC, Tokyo, Japan) in all but Experiments 1, 3, and 9, for which stimuli were presented on an EIZO Flexscan T566 85-Hz monitor (EIZO, Ishikawa, Japan). A head restraint maintained a viewing distance of 57 cm from the NEC monitor and 50 cm from the EIZO monitor, resulting in each pixel intercepting a visual angle of 1.65 arcmin for both monitors. Stimuli were centered on a gray background (8.3° × 8.3°; luminance = 28.2 cd/m^2^), which was centered on the dark monitor screen (luminance = 0 cd/m^2^). They were presented at different, randomly determined locations during each trial. Each location was horizontally and vertically displaced from the center of the screen by between −12.8 and +12.8 arcmin (randomly determined independently for the two dimensions). In all but Experiment 1, a black flanking line segment of the same orientation, size, and shape was located 3.3 arcmin to the left of the test stimuli. A preliminary experiment examining the effect of the black line segment on the visibility of a simultaneously presented white probe is presented in Appendix A. Stimulus presentation and the recording of responses were controlled by MATLAB scripts (MathWorks, Natick, MA) in conjunction with the Psychophysical Toolbox-3 (Brainard, 1997; Kleiner, Brainard, & Pelli, 2007).

### Participants

The 22 voluntary participants were all undergraduate students at Florida Atlantic University, Boca Raton, FL, who were naïve with respect to the purposes of the experiments. Most participated in more than one experiment. All provided signed informed consent approved by the Florida Atlantic University Institutional Review Board, which also exempted the study from specific ethical approval. The research was in accordance with the World Medical Association code of ethics and the tenets of the Declaration of Helsinki.

## Part 1: Mechanisms for stabilizing visibility

### Experiment 1

Preliminary testing indicated that both excitatory and inhibitory interactions affect the *visibility* of the vertical line segments tested in the current study. Previous studies have provided evidence for the distance and contrast dependence of excitatory and inhibitory detector interactions using aligned and misaligned sine gratings. Polat and Sagi (1993) found distance-dependent excitatory and inhibitory effects on the visibility of a Gabor-windowed sine grating (the target) when it was flanked by colinear, orientation-aligned Gabor gratings. The visibility of the target was facilitated for relatively small distances between the flankers and target and was suppressed for larger flanker/target distances. With the same paradigm, Zenger and Sagi (1996) provided evidence for contrast dependence, low-contrast flankers facilitating target visibility, and high-contrast flankers suppressing target visibility. Contrast dependence has also been reported for circular sine gratings surrounded by a sine grating with an orthogonal orientation (Yu, Klein, & Levi, 2002). Yu et al. (2002) found that the perceived contrast of the central grating was increased when the surrounding grating was low in contrast and decreased when the surrounding grating was high in contrast. In psychophysical studies with line segments similar to those of the current study, Kapadia, Westheimer, and Gilbert (2000) found that the tilt illusion was attractive (indicating excitatory interaction) when the line segments were colinear and repulsive (indicating inhibitory interaction) when they were laterally positioned, orthogonal to the orientation of the line segments.

Experiment 1 in the current study determined whether the excitatory and inhibitory interactions that affect the *visibility* of parallel vertical line segments are contrast dependent.

#### Method

**Figure 2.**
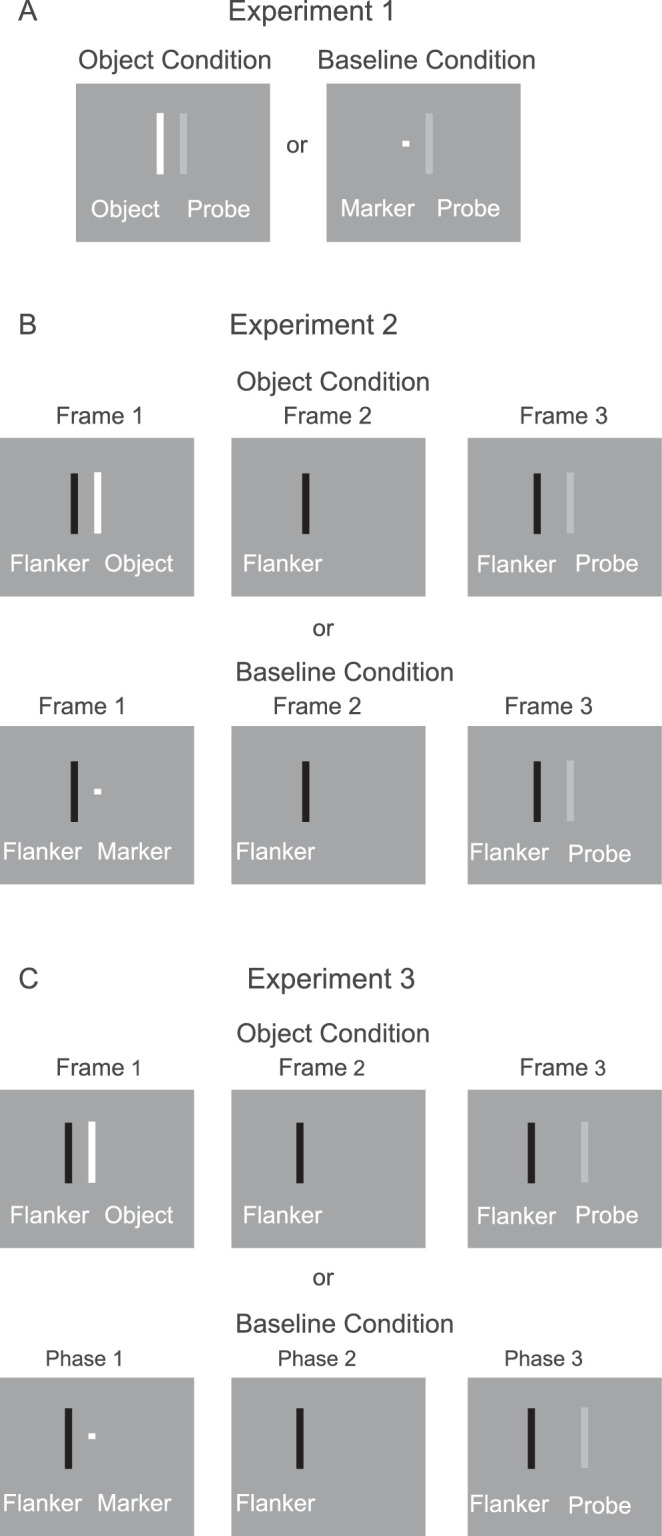
Illustration of stimuli. (**A**) In Experiment 1, the contrast of the object was either high (0.24) or low (0.071). (**B**) In Experiment 2, the object was low in contrast (0.071). (**C**) In Experiment 3, the object was low in contrast (0.071). The contrast of the probe varied between 0.008 and 0.071 in all three experiments.

The stimuli are illustrated in Figure 2A. Two parallel, vertical line segments were presented for half the trials. The line segments were 3.3 × 33 arcmin, and the gap between them was 3.3 arcmin (center-to-center distance was 6.6 arcmin). Both were lighter than the background. The Michelson contrast of the line segment on the left (the “object”) was 0.24 in the high-contrast condition, and 0.07 in the low-contrast condition: Contrast = (Lum_probe_ – Lum_background_)/(Lum_probe_ + Lum_background_. For the other half of the trials, the “object” was replaced by a small 3.3 × 3.3-arcmin white marker (contrast = 0.24). The marker served to minimize failures to detect very low-contrast probes because of uncertainty in their location (Petrov, Verghese, & McKee, 2006). These “marker” trials served as a baseline for the object conditions.

For both object and baseline trials, the contrast of the line segment on the right (the “probe”) was 0.008, 0.016, 0.025, 0.032, 0.040, 0.048, 0.056, 0.064, or 0.071, randomly determined. The object (or marker) and the probe were presented simultaneously for 360 ms. Blocks of 144 order-randomized trials were generated by the orthogonal combination of nine contrast values for the probe, two stimulus conditions (object or marker), and eight repetitions. Eight observers were tested on four such blocks of trials that were presented in Latin square order during each of four testing sessions (two with high-contrast and two with low-contrast objects). After each trial, observers pressed keys on the computer keyboard to indicate whether the probe was visible or invisible.

#### Results

**Figure 3.**
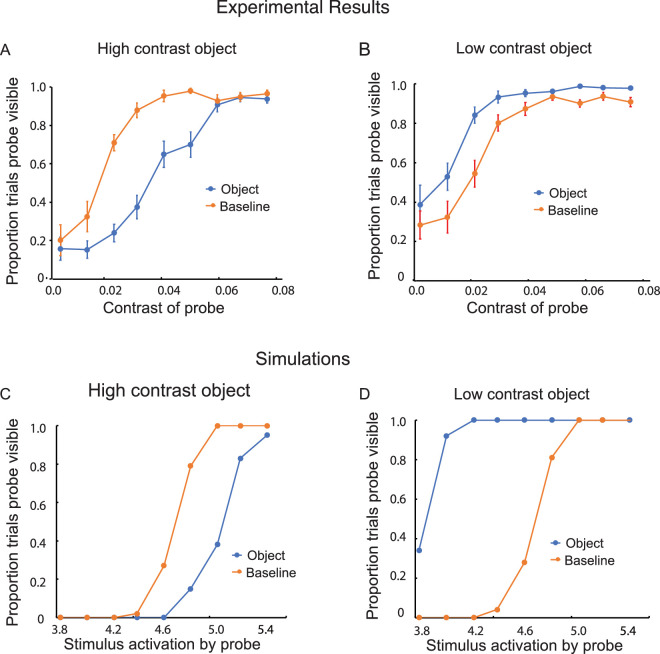
Results of Experiment 1: The effect of a nearby object on judgments of visibility for a variable-contrast probe. (**A**) When the object was high in contrast (0.24), the visibility of the probe decreased relative to the baseline condition: inhibition. (**B**) When the object was lower in contrast (0.071), the visibility of the probe increased relative to the baseline condition: facilitation. (In the baseline condition, the object was replaced by a small dot, the marker.) The vertical line segments denote ±1 *SEM*. (**C**, **D**) Computational simulations of the experimental results based on a dynamic neural field model. The stimulus-initiated activation was 5.0 for the low-contrast object and 10.0 for the high-contrast object. It ranged from 3.8 to 5.4 for the probe.

The probe was visible less often in the object than the baseline condition when the object was high in contrast (Figure 3A). This indication of visibility-suppressing inhibitory interaction was statistically significant, *F*(1, 7) = 54.24, *p* < 0.001. However, for trials with low-contrast objects*,* the probe was visible significantly more often in the object than the baseline condition (Figure 3B). This indication of visibility-enhancing excitatory interaction was statistically significant, *F*(1, 7) = 61.17, *p* < 0.001.

It was found in a separate comparison that the relatively small difference in probe visibility between the baseline trials of the high-contrast and low-contrast conditions was not statistically significant, *F*(1, 7) = 1.74, *p* > 0.05. There was no indication, therefore, that the effects on probe visibility observed in the high-contrast and low-contrast conditions (suppression in one, facilitation in the other) were due to differences between the baseline trials in response criterion and/or spatial uncertainty (Petrov et al., 2006).

#### Discussion

The results for low-contrast objects provide evidence for the existence of lateral excitatory interactions that are the basis for the self-excitation that stabilizes near-threshold visibility. High-contrast objects, which elicit greater stimulus-initiated activation than low-contrast objects, raise detector activation to levels for which visibility-suppressing inhibitory interactions are created. Ideally, a forced-choice procedure would have been used for this experiment in order to eliminate the possibility of response bias affecting the results. This was precluded, however, by effects of memory, distance, and spatial attention that were observed in the experiments that followed. Although differences in response bias remained possible, they do not provide a plausible account of the results. For example, a bias to respond “visible” when an object was present could have been responsible for the greater frequency of “visible” responses for trials with the low-contrast object (compared with the baseline trials). However, it could not at the same time account for the reduced frequency of “visible” responses for trials with the high-contrast object (again compared with the baseline trials). The results of a supplementary experiment measuring the effect of distance on visibility thresholds (Experiment 10) provided further evidence that spatial interaction rather than response bias was responsible for the effects of objects on the visibility of nearby probes.

### Experiment 2

Experiment 1 showed that a low-contrast object can enhance the visibility of a nearby probe. The purpose of Experiment 2 was to determine whether the excitatory effects of a low-contrast object can persist over long temporal intervals during which the object is no longer present. It was determined whether a very low-contrast probe that would otherwise be invisible can become visible as a result of the subthreshold activation from the memory trace laid down during the prior presentation of a low-contrast object.

#### Method

The stimuli are illustrated in Figure 2B. Each trial was composed of three frames during which the line segments were presented at the same randomly determined location. This location changed randomly from one trial to the next. During frame 1 (duration = 360 ms), a vertical, black line segment (luminance = 0 cd/m^2^) was presented to the left of either a white line segment (the object condition) or a small white marker (the baseline condition). The center-to-center distance between the black flanking line and the object (or marker) was 6.6 arcmin (gap = 3.3 arcmin). The contrast of the object and marker was 0.073. During frame 2, only the black flanking line was presented for an interstimulus interval: 107, 200, 307, 400, 507, 600, 707, or 800 ms, randomly determined. It provided a continuing position cue during this otherwise blank interval. During frame 3 (duration = 107 ms), a very low contrast white line (the probe; contrast = 0.008) was presented at the same location as the previously presented object (or marker), along with the flanking black line. The continuous presence of the black flanking line during each trial minimized the possibility that the visibility of the very low-contrast probe line would be adversely affected by uncertainty in its location (Petrov et al., 2006).

Blocks of 96 order-randomized trials were generated by the orthogonal combination of two conditions (object or marker during frame 1), eight durations during frame 2, and six repetitions. Eight observers were tested on four blocks of trials during each of three sessions. After each trial they pressed keys on the computer keyboard to indicate whether the probe was visible or invisible. They were reminded to report whether the probe was visible at the end of the trial, not whether the object was visible at the start of the trial.

#### Results

**Figure 4.**
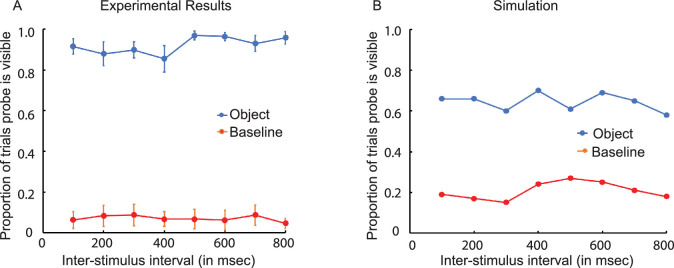
Results of Experiment 2. (**A**) The effect of a low-contrast (0.073) object on the visibility of a very low-contrast probe (0.0008) that was presented at the location previously occupied by the object. The object was presented for 360 ms. A variable-duration interstimulus interval preceded the 107-ms presentation of the probe. (In the baseline condition, the object was replaced by a small dot, the marker.) The vertical line segments denote ±1 *SEM*. (**B**) A computational simulation of the experimental results based on a dynamic neural field model. The stimulus-initiated activation was 5.0 for the object and 4.5 for the probe.

The very low-contrast probe, which was almost always invisible in the baseline condition, was almost always visible in the object condition (Figure 4A). The enhancing effect of the previously presented object on the visibility of the probe was statistically significant, *F*(1, 7) = 280.31, *p* < 0.001. The excitatory effects of the object persisted with little change for up to 800 ms after its removal; the interaction between condition (object vs. baseline) and the interstimulus interval was not statistically significant, *F*(7, 49) = 2.04, *p* > 0.05.

#### Discussion

The visibility of the probe was greatly facilitated by the preceding presentation of a low-contrast object at the same location. This occurred even after a long temporal interval during which the object was no longer present. Had it been above-threshold detector activation that had persisted, the object would have remained visible during the long intervening interval. Because it was not visible, the facilitation of probe visibility where the object was previously located must have been due to the persistence of *subthreshold* activation following the removal of the object. A similar effect of persistence was obtained when the object and probe were in different locations (in Experiment 3, which follows). The latter ruled out the possibility that the persistence effect was an artifact of observers confusing the visibilities of the probe and object because they were presented at the same location.

### Experiment 3

Experiment 3 determined whether the subthreshold activation that persists following the removal of a low-contrast object (as shown in Experiment 2) can facilitate visibility of at locations other than that of the object. This would indicate that subthreshold activation created at one location can affect the visibility when a previously visible object reappears at a new location.

#### Method

The stimuli are illustrated in Figure 2C. As in Experiment 2, each trial was composed of three frames during which the line segments were presented at the same randomly determined location (this location changed randomly from one trial to the next). During frame 1 (duration = 360 ms), a vertical, black line segment (luminance = 0 cd/m^2^) was presented 6.6 arcmin (center-to-center; 3.3 arcmin gap) to the left of either a white line segment (the object condition) or a small white marker (the baseline condition). The contrast of the object and marker was 0.071. During frame 2, only the flanking black line was present for an interstimulus interval of 612 ms. During frame 3, a variable-contrast probe was presented for 106 ms, 6.6 arcmin (center-to-center) to the right of the previous location of the object/marker (the black flanker remains at the same location). The contrast of the probe was 0.008, 0.016, 0.025, 0.032, 0.040, 0.048, 0.056, 0.064, or 0.071, randomly determined.

Blocks of 144 order-randomized trials were generated by the orthogonal combination of nine probe contrast values, two conditions (object or baseline), and eight repetitions. Eight observers were tested on four blocks of trials during each of three sessions. After each trial they pressed keys on the computer keyboard to indicate whether the object was visible or invisible.

#### Results

**Figure 5.**
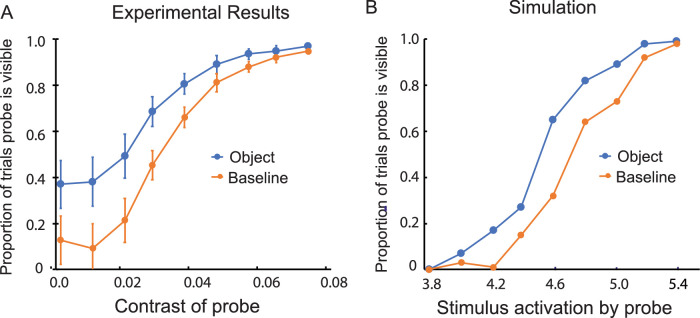
Results of Experiment 3. (**A**) The effect of a low-contrast (0.073) object on the visibility of a variable-contrast probe that was presented 6.6 arcmin (center-to-center) to the right of the location previously occupied by the object. The object was presented for 360 ms, and a blank interval of 614 ms preceded the 107 ms presentation of the probe. (In the baseline condition, the object was replaced by a small dot, the marker.) The vertical line segments denote ±1 *SEM*. (**B**) A computational simulation of the experimental results based on a dynamic neural field model. In the model, the stimulus-initiated activation was 5.0 for the object and ranged from 3.8 to 5.4 for the probe. The center-to-center distance between the object and probe locations was 8 spatial units.

As can be seen in Figure 5A, the previously presented object increased the visibility of a nearby probe; that is, the difference in visibility between in the object and baseline conditions was statistically significant, *F*(1, 7) = 13.17, *p* < 0.01. This provided evidence that subthreshold activation following the removal of an object can restore visibility when it reappears at a new location.

## Part 2: Stabilization: Maintaining visibility over time and space

The preceding experiments provided evidence for two processes that could contribute to the stabilization of low-contrast visibility: (1) visibility-enhancing excitatory detector interactions, the basis for the self-excitation that boosts near-threshold detector activation above the visibility threshold; and (2) short-term memory resulting from above-threshold detector activation creating a memory trace that maintains subthreshold when low-contrast objects are no longer present. The experiments in Part 2 determined whether self-excitation contributes to the stabilization of visibility over time and space when an object is presented multiple times over a sequence of randomly determined locations, with the contrast of an object either kept constant (temporal stability) or gradually decreased (hysteresis).

### Method: Experiments 4 to 7

**Figure 6.**
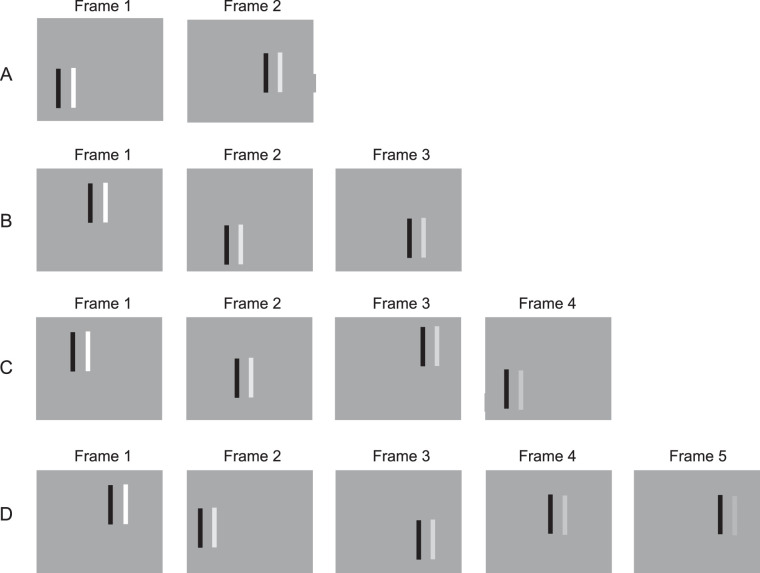
Illustration of stimuli for Experiments 4 through 9. Each trial in these experiments was composed of a variable number of frames during which black and white vertical line segments were presented in a sequence of randomly determined locations. (**A**) Two frames per trial. (**B**) Three frames per trial. (**C**) Four frames per /trial. (**D**) Five frames per trial. The maximum was nine frames per trial. Experiment 4: The contrast of the white line segment was the same during every trial. Experiment 5: Depicted are examples of descending trials; the contrast of the white line segment was decreased during successive frames. For ascending trials, which are not illustrated, the contrast of the white line segment was increased during successive frames. Experiment 6: The same as Experiment 5, except that the contrast during the first frame was presented four times, always at a different randomly determined location. Experiment 7: The same as Experiment 5, except that the contrast during the last frame was presented four times, always at a different randomly determined location. Experiment 8: The same as Experiment 5, except that blank frames were inserted between the frames in which the line segments were presented. Experiment 9: The same as Experiment 5, except that the frame durations were much briefer and the distance between random relocations of the line segments was much larger.

As illustrated in Figure 6, the stimuli again were composed of two parallel, vertical line segments, each 3.3 × 33.0 arcmin (the gap between them was 3.3 arcmin). The line segments were simultaneously presented against a gray background (luminance = 28.2 cd/m^2^). The line segment on the left (the flanker) remained black throughout the experiments (luminance = 0 cd/m^2^). As in the preceding experiments, its purpose was to ensure that the observer could always attend to the location of the variable-contrast, lighter than background line segment on the right, which was sequentially presented (along with the black flanker) at a sequence of randomly determined locations.

#### Frame durations

The first frame of each trial was 614 ms in duration in order to ensure that there would be sufficient time for observers to attend to the object (its location was uncertain) and determine whether it was visible or not. All of the frames following the first were 360 ms in duration. The interframe intervals were 0 ms.

#### Randomized locations

During each trial, the black flanker and the white object were presented at a succession of different, randomly determined locations. Each location was horizontally and vertically displaced from the center of the screen by between −12.8 and +12.8 arcmin (randomly determined independently for the two dimensions). The mean distance between successive relocations was 14.3 arcmin.

#### The black flanker

The presence of the black flanking line marked the beginning and end of each trial. Were the black line not presented alongside the object, there would have been ambiguity at the start of every trial: “Has the trial begun and the object is invisible or has the trial not yet begun?” Similarly, there would have been ambiguity at the end of every trial: “Did the object become invisible before the end of the trial or did the trial end while the object was still visible?”

#### Procedure

After each trial, observers pressed keys on the computer keyboard to indicate whether the object was visible or invisible at the start of the trial and then whether there was a change from visible to invisible, or vice versa, anytime during the trial. There was no requirement to respond as quickly as possible.

### Experiment 4

Experiment 4 tested the stabilization of near-threshold visibility over time and space with a bistable object (line segment). Sometimes it was visible at the start of the trial, and sometimes it was initially invisible. This initial bistability was assumed to be the result of activation either being boosted over the detection threshold by self-excitation or remaining below the detection threshold when random fluctuations in activation prevented the eliciting of self-excitation. The experiment determined whether near-threshold visibility, when established via self-excitation, would be stabilized when the same object was repeatedly presented over a sequence of randomly determined locations. Stabilization of the visible state would be indicated by a low rate of switching from visibility to invisibility. Stability of the invisible state would be indicated by a low rate of switching from invisibility to visibility.

#### Method

The experiment followed the methodology developed for assessing the temporal stability of the vertical and horizontal motion patterns perceived for motion quartets (Hock, Schöner, & Voss, 1997). After their first 614-ms presentation at a randomly determined location, the black flanker and white object were presented again for 360 ms at a succession of randomly determined locations. There were 2, 3, 4, 5, 6, 7, 8, or 9 presentations per trial. The second presentation provided the first opportunity of a visibility-to-invisibility or invisibility-to-visibility switch. The contrast of the object (0.041) was selected so that it would be visible at the start of approximately half the trials.

Blocks of 96 randomly ordered trials were formed by 12 repetitions of the eight trial types with between two and nine presentations. Four blocks of trials were tested during each of four testing sessions. Eight observers indicated, after each trial, whether the object was visible or invisible at the start of the trial and then whether there was a switch to the alternative at any time during the trial. The stimulus sequences for Experiment 4 to 9 are illustrated in Figure 6.

#### Results

**Figure 7.**
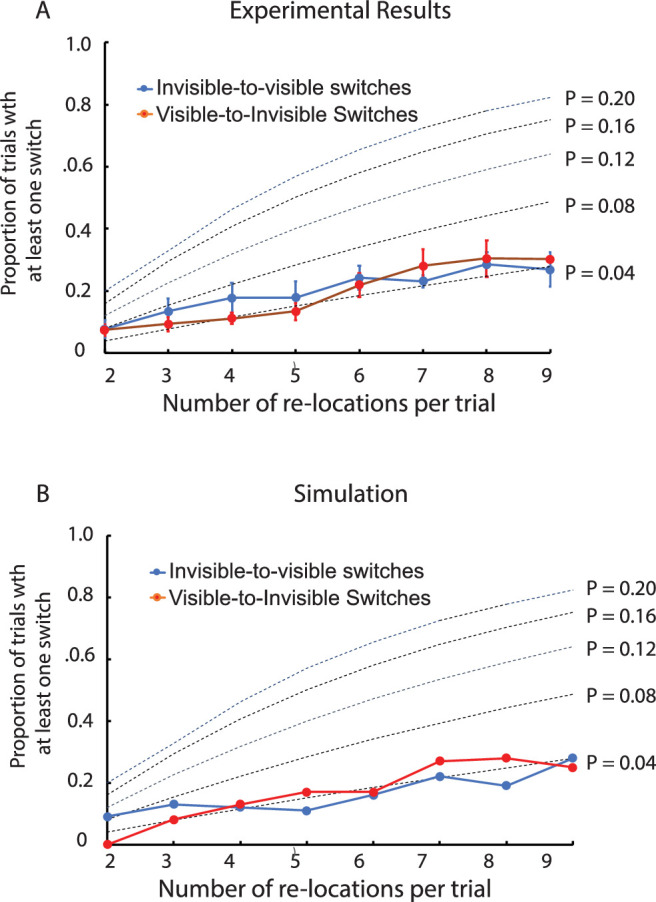
Results of Experiment 4. (**A**) The proportion of trials during which there was at least one switch from visibility to invisibility or from invisibility to visibility, both as a function of the number of 360-ms relocations of a low-contrast (0.041) object following its initial 614-ms presentation. The object was visible for 62% and invisible for 38% of the initial presentations of the object. The dotted lines represent the calculated probability of a switch during each relocation of the object, assuming switching probability remained constant across all the relocations. The vertical line segments denote ±1 *SEM*. (**B**) A computational simulation of the experimental results based on a dynamic neural field model. In the model, the stimulus-initiated activation was 4.7. For the initial presentation of the object, visibility was indicated by the model for 62% of the trials and invisibility for 38% of the trials.

The object was reported as visible during its initial presentation for 62% of the trials; the proportions of trials during which there was at least one switch from visible to invisible are presented in Figure 7A. The object was reported as invisible during its initial presentation for 38% of the trials; the proportions of trials during which there was at least one switch from invisible to visible are also presented in Figure 7A. In both cases, the results are reported as a function of the number of times the object was presented in each trial. The similarity of the visible-to-invisible and invisible-to-visible switching rates was consistent with invisibility judgments being based on the absence of visibility.

Presented along with these data are broken lines indicating the likelihood of at least one switch occurring anytime during a trial with *N* presentations of the object calculated based on the assumption that the probability (*p*) of a switch during each presentation remained constant throughout the trial. That is, *Probability* {1 or more switches} = 1 – (1 – *p*)*^N–1^*, where (1 – *p*) is the probability of there not being a switch in each of the *N* – 1 presentations during a trial. The calculated functions closest to the empirical results were obtained for *p* = 0.04. The likelihood of a switch during any of the presentations following the first was thus very low for both visible-to-invisible and invisible-to-visible switches.

#### Discussion

The results established for the first time, to the best of our knowledge, that near-threshold visibility can be stabilized over time and space. We observed that the visibility of a low-contrast object, established during its first presentation, was maintained for up to 3 seconds across as many as eight unpredictable relocations of the object. When the object was invisible at the start of a trial, invisibility remained for up to 3 seconds, not because invisibility is a stable state but because invisibility was reported in the absence of visibility.

The bistability observed at the start of each trial (the object was visible for 62% and invisible for 38% of the trials) was likely due to the observers’ perceptual sensitivity drifting over the course of multiple trials. When established, however, the likelihood of a switch between visibility and invisibility was only 0.04. This temporal stability was attributable to self-excitation increasing the activation gap between the visible and invisible perceptual states, thereby decreasing susceptibility to the effects of drifting visual sensitivity and random fluctuations in detector activation. That is, only relatively large (and therefore relatively rare) fluctuations would have been sufficient to produce a switch between visibility and invisibility. Were it not for self-excitation, the probability of a switch during each frame after the first would have been similar to the probabilities of visibility and invisibility during frame 1 (i.e., close to 0.5). Experiment 7 confirmed that stochastic fluctuations affect switching in the current paradigm.

### Experiment 5

The preceding experiment provided evidence for the maintenance of visibility for a low-contrast object, despite unpredictable changes in the location of the object. The purpose of Experiment 5 was to determine whether this stabilization of visibility would extend to low-contrast objects that were changing in contrast. This would be indicated by evidence for hysteresis when comparing visibility for descending trials with gradually decreasing contrast and ascending trials with gradually increasing contrast.

#### Method

Ascending trials began with the lowest contrast (0.008). Contrast was then increased in discrete 0.008 steps during successive presentations, each step at a different randomly determined location. Descending trials began with the highest contrast value (0.073). Contrast was then decreased in discrete 0.008 steps during successive presentations, again with each step at a different randomly determined location.

##### The modified method of limits

If the classical method of limits had been used, the full range of contrast values would have been presented during every ascending and every descending trial. Observers would have been required to respond in the midst of the trial, as soon as they perceived a switch between invisible and visible (or vice versa). Given variability in decision and response times, the determination of when a switch occurred would be uncertain. The *modified* method of limits was used instead (Hock, Kelso, & Schöner, 1993; Hock & Schöner, 2010). The method determines when switches occurred without requiring the observer to respond in the midst of an ascending or descending sequence, and without concern for how quickly a response was executed. Accordingly, the contrast of the object was gradually decreased or increased by a *variable number of steps* during each trial, so the final end-of-trial contrast varied from trial to trial.

A change in visibility was expected to be relatively infrequent for trials with just a few steps. For trials with more steps, the likelihood of visibility (or invisibility) persisting for the entire trial would be expected to decrease as the number of steps was increased. Hysteresis would then be indicated when an object was more likely to be visible for a particular end-of-trial contrast value when it was reached via a descending compared with an ascending sequence of contrast changes.

##### Design

There were eight kinds of ascending trials (end-of-trial contrasts were 0.017, 0.025, 0.033, 0.041, 0.049, 0.057, 0.065, 0.073, or 0.081) and eight kinds of descending trials (end-of-trial contrasts were 0.008, 0.017, 0.025, 0.033, 0.041, 0.0490, 0.057, 0.065, or 0.073). The sequences for descending trials are illustrated in Figure 6. For brief descending trials with an end-of-trial contrast of 0.017, the sequence of presentations was 0.008–0.017, each at a randomly determined location. The sequence of presentations for slightly longer descending trials with an end-of-trial contrast of 0.025 was 0.008–0.017–0.025, each at a randomly determined location, and so on, up to the longest descending trials: 0.008–0.017–0.025–0.033–0.041–0.049–0.057–0.065–0.073, each at a randomly determined location. The 16 trials thus generated (eight descending and eight ascending) were repeated six times, forming blocks composed of 96 randomly ordered trials. Eight observers were tested on four blocks of trials in each of three sessions.

##### Analysis of hysteresis

The analysis included only ascending trials for which the object was invisible during its initial presentation and only descending trials for which the object was visible during its initial presentation (a high percentage of the trials in both cases). For ascending trials, the first opportunity for an invisible-to-visible switch occurred when contrast was increased by a single step (from 0.008 to 0.017). For descending trials, the first opportunity for a visible-to invisible switch occurred when contrast was decreased by a single step (from 0.071 to 0.063). The results were statistically analyzed by comparing visibility for end-of-trial contrast values that were common to the ascending and descending trials (between 0.017 and 0.063).

#### Results and discussion

**Figure 8.**
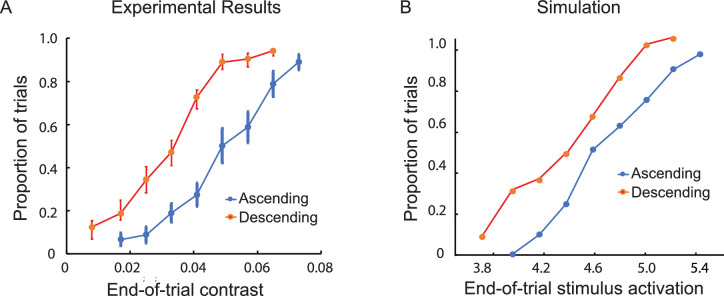
Results of Experiment 5. (**A**) The proportion of ascending trials during which there was at least one switch from invisibility to visibility and the proportion of descending trials during which the object remained visible throughout, both as a function of the contrast of the object at the end of a trial. When the initial contrast of the object was very small (0.008), it was invisible during the initial 614-ms presentation of each ascending trial. Its contrast then increased in steps of 0.008, ending after a variable number of steps (and, thus, a different end-of-trial contrast). When the initial contrast of the object was greater (0.073), it was visible during the initial 614-ms presentation of each descending trial. Its contrast then decreased in steps of 0.008 at each 360-ms relocation of the object, ending after a variable number of steps (and, thus, a different end-of-trial contrast). The vertical line segments denote ±1 *SEM*. (**B**) A computational simulation of the experimental results based on a dynamic neural field model. The stimulus-initiated activation of the object ranged from 3.8 to 5.4.

Visibility was greater for end-of-trial contrast values that were reached by gradually decreasing compared with gradually increasing contrast (Figure 8A). This evidence for hysteresis was statistically significant, *F*(1, 7) = 30.08, *p* < 0.001. Thus, visibility was maintained for descending trials despite reductions in contrast to levels that would otherwise have resulted in invisibility. If there were no excitation-elicited activation associated with the descending trials, visibility would have been equally likely for the descending and ascending trials ending at the same contrast; there would have been no evidence for hysteresis. Obtaining hysteresis provided direct evidence for the linkage between successive relocations of an object; that is, when an object was visible at one location, the likelihood was increased that it would remain visible when it was relocated.

Visibility for descending trials would be expected to continue with each relocation of the object as long as the combined stimulus-induced activation and self-excitation was sufficient to exceed the threshold for visibility. However, the stochastic switching between visibility and invisibility observed in Experiment 4 suggests that switches induced by random fluctuations in detector activation may have reduced the full range of perceptual bistability.

### Experiment 6

This experiment tested for the asymmetry inherent in the proposition that visibility is stabilized by self-excitation, which boosts detector activation above the visibility threshold. Invisibility is represented, in contrast, by the absence of self-excitation that would boost activation above the visibility. The purpose of Experiment 6 was to confirm this asymmetry by providing evidence for asymmetrical effects of prior adaptation. Adaptation occurs when stimulus input induces above-threshold activation. It then lowers stimulus-induced activation (putatively by weakening the neural connectivity that mediates stimulus input). Prior adaptation (by repeatedly presenting the initial contrast level of each trial) was therefore expected to affect descending trials, which begin with a visible contrast level, but not ascending trials, which begin with an invisible contrast level.

#### Method

The starting contrast values of descending and ascending trials were presented either once or four times, always at different randomly determined locations. Contrast was then decreased or increased during successive representations, as in the preceding hysteresis experiment. Blocks of 96 order-randomized trials were generated by the orthogonal combination of 16 contrast sequences (eight end-of trial contrast values for descending and ascending trials), two conditions for the initial contrast value (presented either once or four times), and three repetitions. Eight observers were test on four blocks of trials during each of three sessions.

#### Results

**Figure 9.**
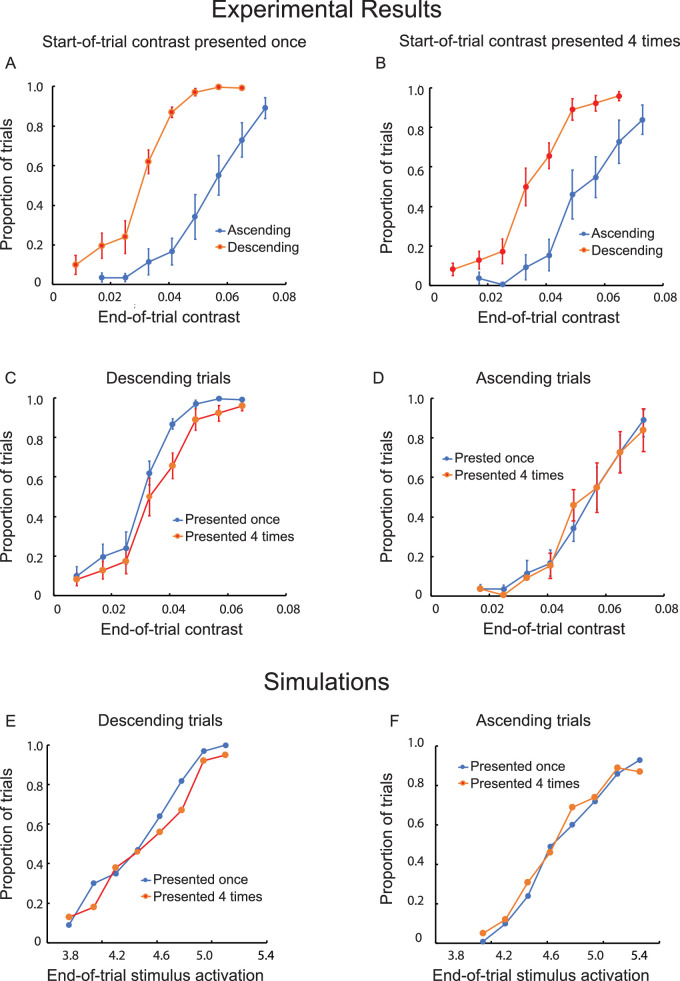
Results of Experiment 6. The proportion of ascending trials during which there was at least one switch from invisibility to visibility and the proportion of descending trials during which the object remained visible throughout, both as a function of the contrast of the object at the end of the trial. The start-of-trial contrast was presented once (**A**) or four times (**B**). The contrast of the object was increased or decreased in steps of 0.008 at each of its randomly determined relocations, ending after a variable number of steps (and, thus, a different end-of trial contrast). One versus four start-of-trial presentations are compared for descending (**C**) and ascending (**D**) trials. The vertical line segments denote ±1 *SEM*. Computational simulations for descending (**E**) and ascending (**F**) trials are based on a dynamical neural field model. Stimulus-initiated activation for the object ranged from 3.8 to 5.4.

As in Experiment 5, the difference in visibility between the descending and ascending trials (i.e., the hysteresis effect) was statistically significant when there were no repetitions of the initial contrast value, *F*(1, 7) = 50.77, *p* < 0.001 (Figure 9A). Although reduced in magnitude, the hysteresis effect also was statistically significant when there were four repetitions of the initial contrast value, *F*(1, 7) = 36.04, *p* < 0.001 (Figure 9B). As can be seen in Figures 9C and 9D, the reduction in hysteresis was due entirely to adaptation reducing visibility for the descending trials. The difference between trials with and without repetitions of the initial contrast was statistically significant for the descending trials, *F*(1, 7) = 16.14, *p* < 0.005, but not for the ascending trials, *F*(1, 7) = 0.01, *p* > 0.05.

#### Discussion

Prior adaptation provided evidence for the asymmetric stabilization of visibility and invisibility. That is, repetitions of the initial contrast increased switching from visibility to invisibility for descending trials but did not affect invisible-to-visible switches for the ascending trials. There was no above-threshold detector activation to adapt for the ascending trials.

Symmetrical increases in switching between *competing* perceptual alternatives are typically reported in experiments assessing hysteresis (e.g., Hock et al., 1993; Gephstein & Kubovy, 2004; Gori, Giora, & Pedersini, 2008). In contrast, the asymmetrical adaptation-induced reduction in hysteresis obtained in the current experiment indicates that the perceptual alternatives, visibility and invisibility, do not compete. Invisibility is simply the absence of visibility.

### Experiment 7

In the preceding hysteresis experiments, the number of presentations per trial of the object varied from trial to trial, resulting in the contrast level at the end of each trial also varying from trial to trial. Some trials ended after just a few contrast decrements or increments; others ended after many contrast decrements or increments. In Experiment 7, end-of-trial contrast levels, when reached, were repeated three times. The end-of-trial repetitions provided additional opportunity for adaptation and random fluctuations to reduce the above-threshold activation that stabilizes visibility for descending trials, as well as the additional opportunity for the occurrence of fluctuation-induced switches between visibility and invisibility during ascending trials.

#### Method

The end-of-trial contrast values of descending and ascending trials were presented either once or four times, each at a different randomly determined location. Blocks of 96 order-randomized trials were generated by the orthogonal combination of 16 contrast sequences (eight end-of-trial contrast values for ascending and ascending trials), two conditions for the end-of-trial contrast (presented once or four times), and three repetitions. Eight observers were test on four blocks of trials during each of three sessions.

#### Results

**Figure 10.**
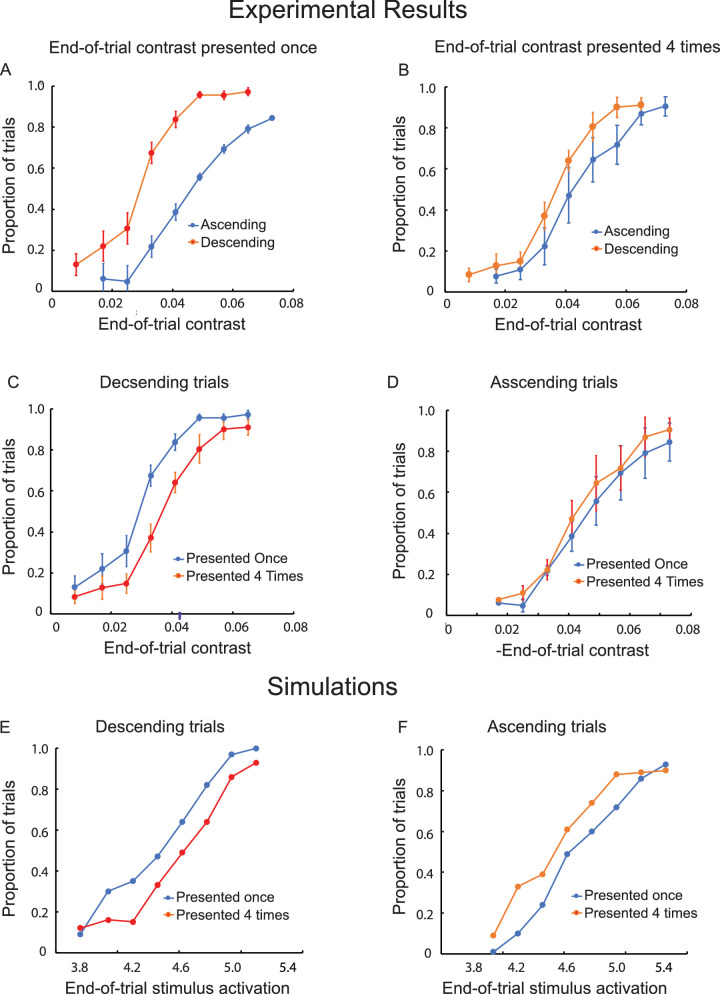
Results of Experiment 7. The proportion of ascending trials during which there was at least one switch from invisibility to visibility and the proportion of descending trials during which the object remained visible throughout, both as a function of the contrast of the object at the end of the trial. The end-of-trial contrast was presented once (**A**) or four times (**B**). One versus four end-of-trial presentations are compared for descending (**C**) and ascending (**D**) trials. The vertical line segments denote ±1 *SEM*. Computational simulations for descending (**E**) and ascending (**F**) trials are based on a dynamical neural field model. Stimulus-initiated activation for the object ranged from 3.8 to 5.4.

As in the preceding experiments, the difference in visibility between the descending and ascending trials (i.e., the hysteresis effect) was statistically significant, but only when the end-of-trial contrast was presented once, *F*(1, 7) = 14.07, *p* < 0.01 (Figure 10A). The hysteresis effect was not statistically significant when there were four repetitions of the end-of-trial contrast, *F*(1, 7) = 2.66, *p* > 0.05 (Figure 10B). This reduction in hysteresis was due to switching during the end-of-trial repetitions for both the descending and ascending trials. The difference between the descending trials with and without end-of-trial repetitions was statistically significant, *F*(1, 7) = 54.93, *p* < 0.001, providing evidence for the occurrence of switching during the end-of-trial repetitions (Figure 10C). Although small, the difference between the ascending trials with and without end-or-trial repetitions was statistically significant, *F*(1, 7) = 5.98, *p* < 0.00, again evidence for the occurrence of switching during the end-of-trial repetitions (Figure 10D). The effect of repeating the end-of-trial contrast values was significantly greater for the descending than the ascending trials, *t*(7)= 4.74, *p* < 0.01.

#### Discussion

The greater frequency of visible-to-invisible switches during the end-of-trial repetitions of the descending trials was likely due, at least in part, to additional adaptation-reduced activation during the end-of-trial repetitions. In contrast, the invisible-to-visible switches during the end-of-trial repetitions for ascending trials could not have been the result of adaptation-reduced activation. Even if there was sufficient activation to adapt, it would have reduced rather than increased invisible-to-visible switching by leaving detector activation further from the level at which self-excitation would be engaged. The invisible-to-visible switches during the end-of-trial repetitions could only have been the result of fluctuations in detector activation.

## Part 3: Linking successive presentations

The evidence for temporal stability and hysteresis in the preceding experiments showed that the visibility of low-contrast objects presented at a succession of locations is not determined independently at each location. The final two experiments show that successive re-presentations of low-contrast objects are linked by subthreshold activation.

### Experiment 8

The results of Experiments 2 and 3 indicated that the detector activation produced by a low-contrast object affected the visibility of a probe for up to 800 ms after the object was no longer present. In these experiments, the effects of persisting subthreshold activation were obtained when the object and probe were presented just once, with no changes in their location. In contrast, Experiment 8 tested for the persisting effects of above-threshold activation when the object was repeatedly presented at different, *unpredictable* locations. It was determined whether hysteresis would be obtained despite the insertion of long blank intervals between successive re-locations of the object. Nothing was visible during these blank intervals, so evidence for hysteresis would be informative with regard to how the persistent subthreshold activation “bridges” the blank intervals and restores visibility when the object reappears at an unpredictable location.

#### Method

The stimuli and design were as in Experiment 5. Hysteresis was measured without the start-of-trial or end-of-trial repetitions of Experiments 6 and 7 The distinctive feature of the current hysteresis experiment was the insertion of 614-ms blank intervals between relocations of the black flanker and white object, the contrast of the white object increasing or decreasing with each relocation. Blocks of 96 randomly ordered trials were generated by the six repetitions of the 16 contrast sequences (eight end-of-trial ascending and ascending trials). Four blocks of trials were tested during two testing sessions.

#### Results

**Figure 11.**
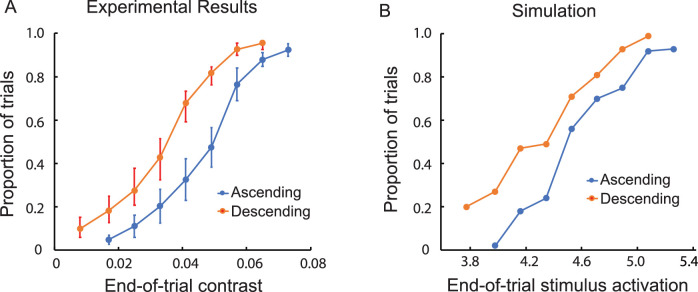
Results of Experiment 8. (**A**) The proportion of ascending trials during which there was at least one switch from invisibility to visibility and the proportion of descending trials during which the object remained visible throughout, both as a function of the contrast of the object at the end of a trial. As in the preceding experiments, contrast was increased or decreased in 360-ms steps of 0.008 as the object was presented over a succession of randomly determined locations. Unlike the preceding hysteresis experiments, 614-ms blank intervals were inserted between successive presentations of the object. The vertical line segments denote ±1 *SEM*. (**B**) Computational simulations based on a dynamic neural field model; the stimulus-initiated activation of the object ranged from 3.8 to 5.4.

Despite the insertion of 614-ms blank intervals, the difference in visibility between the descending and ascending trials (i.e., the hysteresis effect) remained statistically significant, *F*(1, 7) = 17.54, *p* < 0.005 (Figure 11A). This provided evidence for the effects of above-threshold detector activation persisting for relatively long temporal intervals following the removal of the object.

#### Discussion

If visibility had been continuous during the blank intervals (as might occur for much briefer blank intervals), it would have indicated that the hysteresis was the result of above-threshold detector activation itself carrying forward in time, across the intervening blank intervals. However, as in Experiments 2 and 3, this possibility was ruled out by discontinuities in visibility; nothing was present during the blank intervals. The hysteresis was instead attributable to the above-threshold activation, which is responsible for visibility when the object is present, “pre-shaping” the visibility of the object in the immediate future. Because the location where the object would reappear was unpredictable, pre-shaping via the spread of subthreshold detector activation must have occurred over a region surrounding the previous location of the object.

### Experiment 9

The 360-ms presentations of the object in the preceding experiments made it possible for observers to re-fixate on the object following each of its relocations. If this were the case, the pre-shaping by subthreshold activation that was observed in Experiment 8 would be limited to foveal detectors. The purpose of Experiment 9 was to assess hysteresis while ruling out this possibility by making it impossible for observers to re-fixate on the object during its successive, unpredictable relocations. In contrast with preceding experiments, the duration of the object at each relocation was reduced to 116 ms, and the average distance between relocations was doubled (now 28.6 arcmin). The time between relocations remained at 0 ms. Blocks of 96 randomly ordered trials were generated by six repetitions of the 16 contrast sequences (eight ascending and eight ascending trials).

The contrasts in this experiment were taken from this set: 0.008, 0.016, 0.025, 0.032, 0.040, 0.048, 0.056, 0.064, and 0.071. Three blocks of trials were tested during each of two testing sessions.

#### Results

**Figure 12.**
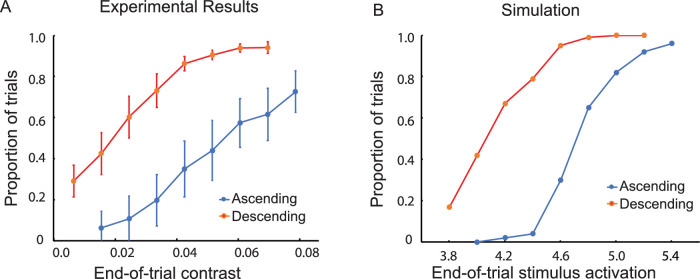
Results of Experiment 9. (**A**) The proportion of ascending trials during which there was at least one switch from invisibility to visibility and the proportion of descending trials during which the object remained visible throughout, both as a function of the contrast of the object at the end of a trial. Contrast was increased or decreased in steps of 0.007 as the object was presented over a succession of randomly determined locations. The duration of each presentation was reduced to 116 ms, and the average distance between successive relocations of the object was doubled compared with the preceding experiments. The vertical line segments denote ±1 *SEM*. (**B**) A computational simulation based on a dynamic neural field model; stimulus-initiated activation for the object ranged from 3.8 to 5.4.

Despite impossibility of re-fixation at successive locations of the object, the difference in visibility between the descending and ascending trials (i.e., the hysteresis effect) was statistically significant, *F*(1, 5) = 36.06, *p* < 0.005 (Figure 12A). This indicated that the subthreshold activation that linked successive relocations of the object was not limited to detectors in the fovea but instead were spread over a relatively wide retinal region surrounding each location of the object.

## Part 4: General discussion and computational simulations

**Figure 13.**
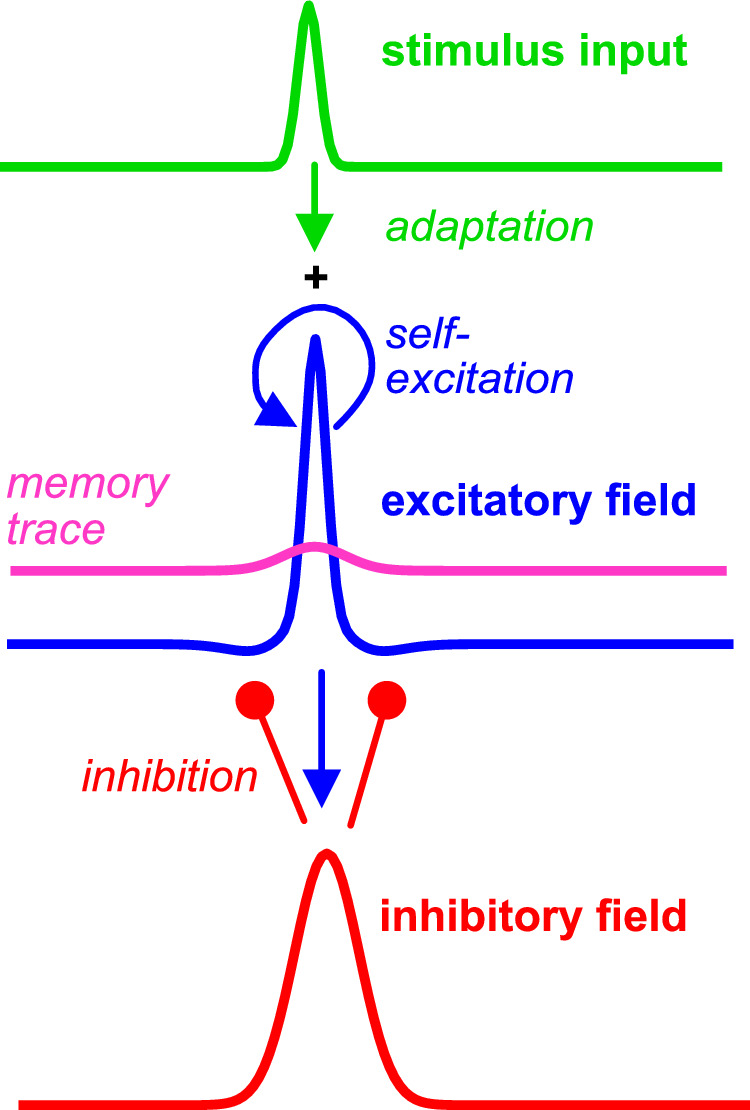
A sketch of the dynamic neural field model. The excitatory neural field (blue) receives input from the stimulus (green) that is localized in visual space. Local recurrent connectivity of the excitatory field leads to self-excitation where activation is above threshold. Activation above threshold also lays down a memory trace (pink) that locally increases the resting level (non-stimulus activation state). At the same time, activation above threshold leads to adaptation, the weakening of input strength given the same stimulus. The excitatory field provides input to the inhibitory field (red), which makes recurrent inhibitory connections onto the excitatory field. These become active only when the inhibitory field has reached threshold.

The experiments reported in this article have provided evidence that the visibility of near-threshold, low-contrast objects is stabilized over time and space as a result of self-excitation boosting stimulus-induced activation above the visibility threshold and short-term memory. Simulations of the results of these experiments, which follow, are based on a neural dynamic model that incorporates self-excitation and a simple model of short-term memory. The model takes into account the potentially destabilizing effects of recurrent inhibition and adaptation (Figure 13).

The mathematical details of the dynamic neural field model, as well as parameter values and information about the performed numerical simulations, are provided in Appendix B. The model provides a neural process account; that is, it simulates how neural activation evolves in time under the influence of inputs that reflect the experimental stimuli and their time structure. The activation variables give rise to “observations” of the state of the model (described in Appendix B) which can be compared to the responses participants give in the different experimental tasks. Because activation fluctuates under the influence of neural noise, the model accounts for probabilities (of detection, of switching, etc.) by generating variable observations from trial to trial. We aimed at a qualitative rather than a quantitative account for all experimental data from a single parameter set, an achievement for such a neural process account.

### Dynamic neural fields

Cortical maps representing visual objects can be modeled by neural activation fields that are defined over visual (retinal) space (Wilson & Cowan, 1973; Amari, 1977; Adini, Sagi, & Tsokyks, 1997; Gerstner, Kistler, Naud, & Paninski, 2014, Chapter 18; Schöner, Spencer, & DFT Research Group, 2016). Activations in these maps evolve in time according to neural dynamics that capture how the firing patterns in populations of neurons are driven by sensory input and recurrent neural connectivity. In neural field models, the spiking mechanism is replaced by a sigmoidal threshold function. Neural fields come in pairs of excitatory and inhibitory fields.

Stimulus input activates the excitatory field. The excitatory field drives the inhibitory field, which recurrently inhibits the excitatory field. Localized suprathreshold activation in the excitatory field indicates that a localized stimulus has been detected (i.e., is visible). The dynamics of neural fields provide two sources of stabilization for such detections: (1) Recurrent excitatory connections within the excitatory field generate self-excitation as local, stimulus-initiated activation approaches the threshold for visibility, and (2) The resulting suprathreshold activation builds a memory trace in the manner of a temporal low-pass filter. The memory trace effectively raises the local resting level of the field. When the stimulus input is removed, subthreshold activation remains (Figure 1B).

The first mechanism is central to the theory. A numerical simulation of the model shown in Figure 1A illustrates how weak input from a localized stimulus induces subthreshold activation at the corresponding location in the field. The simulation shown in Figure 1B shows how stronger input pushes stimulus-initiated activation closer to threshold, engaging self-excitation. At this point, the subthreshold stimulus-induced activation ceases to be a stable neural state due to the detection instability (Schöner et al., 2016, Chapter 2). Activation increases toward an above-threshold peak that is supported by self-excitation and the formation of a memory trace (Figure 1B). We hypothesize that this is the neural activation state that leads to visibility of the stimulus.

Even for the weaker level of stimulus input in Figure 1A, however, two alternative neural activation states may be stable, one above and the other below threshold. Toward higher input levels, this bistable regime is delimited by the detection instability in which the subthreshold stable state is lost. Toward lower input levels, the bistable regime is delimited by the reverse detection instability in which the above threshold peak is lost. Which of the two neural activation states is realized when a stimulus is first presented depends on random fluctuations and prior levels of activation. Experiments 4 to 9 probed such bistability through stochastic switching and perceptual hysteresis.

**Figure 14.**
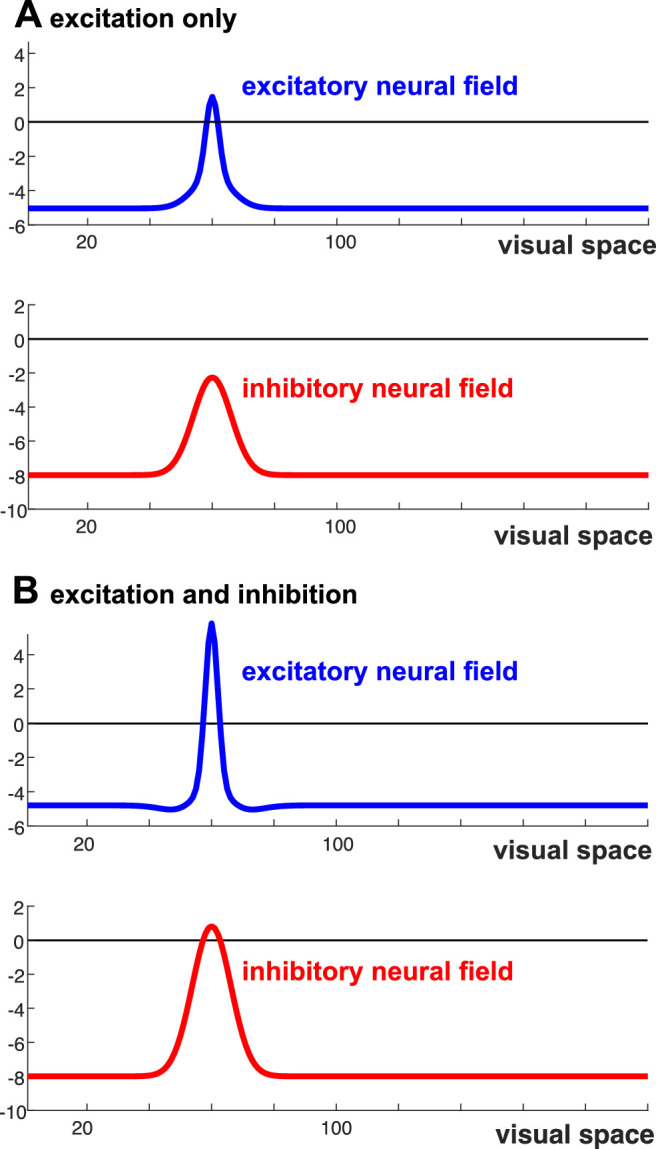
The excitatory and inhibitory neural fields are shown in a stable state when a stimulus-initiated activation is relatively weak (**A**) or stronger (**B**). Moderate (**A**) or strong (**B**) levels of stimulus input are present. (A) An activation peak builds at the stimulus location in the excitatory field, but activation in the inhibitory field remains below threshold. (**B**) Activation peaks form in both fields.

Stabilized visibility is opposed by two other mechanisms. First, recurrent inhibition is engaged when activation in the excitatory field is strong enough to bring the inhibitory field close to its threshold. Figure 14 illustrates the excitatory and inhibitory fields in two cases. In Figure 14A, only the excitatory field has an above-threshold peak of activation in response to stimulus input of weak or moderate strength. Activation in the inhibitory field is below threshold, so there is no inhibitory influence on the excitatory field. In Figure 14B, the excitatory field has a stronger activation peak in response to stronger stimulus input. This provides sufficient input to the inhibitory field for it to develop above-threshold activation. Inhibition affects the excitatory field, limiting the further growth of activation in that field for further increases of input strength. This inhibitory influence from high-contrast visible objects was directly observed in Experiment 1. Second, adaptation, which is observed across all sensory systems, weakens stimulus input whenever stimulus input has successfully induced above-threshold activation. Sufficient self-excitation and memory trace may compensate for the effects of adaptation. Experiment 6 observed the influence of adaptation on above-threshold activation states (for visible objects) and confirmed the absence of such influence for subthreshold activation states (for invisible objects).

### Excitatory and inhibitory neural interactions

Experiment 1 probed how low-contrast and high-contrast objects affect detection at neighboring locations. As illustrated in Figure 14, the model predicts that high-contrast objects engage inhibition as well as self-excitation, to the net effect of increasing the threshold for the detection of a probe (decreasing its visibility) in the vicinity of the object. Low-contrast objects engage only self-excitation and thus lower the threshold for the detection of a probe (increasing its visibility) in their vicinity. The model successfully captures these experimental observations. The transition from facilitation to inhibition with increasing contrast of an object is consistent with the generic coupling structure of neural field models (Figures 3C and 3D). If inhibition were only present in the form of feed-forward inhibitory side bands, this transition would not be predicted.

This and all remaining simulations were obtained from a single set of parameter values of the model (listed in Appendix B), the different simulations differing only in the stimulus conditions of the different experiments. All experiments beyond Experiment 1 entailed only low-contrast objects for which the effects of self-excitation and short-term memory predominated. However, because the distinction between excitatory and inhibitory interactions in Experiment 1 is foundational for the theoretical account of the results obtained in the subsequent eight experiments, it was important to rule out the possibility that the results of Experiment 1 were instead due to response bias. Experiment 10 was conducted in order to confirm that the effects on visibility observed in Experiment 1 entailed spatial interaction rather than response bias.

### Experiment 10

Object contrast values and frame durations were the same as in Experiment 1, but, unlike that experiment, the spatial gap between the object (or marker) and the probe was varied. During each trial the stimulus was repeatedly presented in a different randomly determined location. After each presentation, observers reduced the contrast of the probe for the presentation that followed, with these contrast adjustments continuing until the probe was no longer visible. The contrast at the point of disappearance constituted the visibility threshold for that trial.

**Figure 15.**
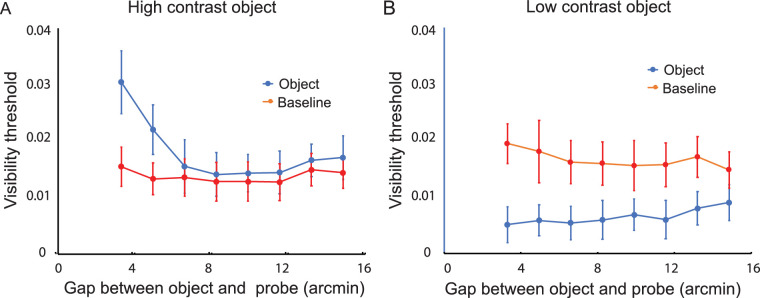
Results of Supplementary Experiment 10: The effect of a nearby object on visibility thresholds of a probe located a variable distance from the object. Visibility thresholds were determined by reducing the contrast of the probe until it was no longer visible. (**A**) When the object was high in contrast (0.24), the visibility of the probe was decreased relative to the baseline condition. (**B**) When the object was lower in contrast (0.071), the visibility of the probe increased relative to the baseline condition The vertical line segments denote ±1 *SEM*.

Consistent with the results of Experiment 1, visibility thresholds were higher for the object than the baseline trials in the high-contrast condition (Figure 15A). This indication of suppressed probe visibility was statistically significant, *F*(1, 5) = 12.10, *p* < 0.02. Also consistent with the results of Experiment 1, visibility thresholds were lower for the object than the baseline trials in the low-contrast condition (Figure 15B). This indication of facilitated probe visibility was statistically significant, *F*(1, 5) = 56.93, *p* < 0.001. Finally, the effect of both high-contrast and low-contrast objects on probe visibility decreased as the size of the gap between them was increased. The interaction between condition (object vs. baseline) and gap distance was statistically significant for the high-contrast object, *F*(7, 35) = 10.56, *p* < 0.001, and the low-contrast object, *F*(7, 35) = 2.34, *p* < 0.05.

The effects of object and probe distance on the facilitation and suppression of probe visibility were as would be expected if the effects were indeed the result of spatial interaction. Although response bias was possible, it is not plausible that it would change over retinal distances smaller than 16 arcmin.

### Short-term memory

Experiment 2 showed that a visible low-contrast object can facilitate the visibility of probes presented at the same location as the object, long after the object has been removed. This persistence of the excitatory effects of an object was attributed to the retention of subthreshold activation. If it were above-threshold activation that was retained, the object would have remained visible even when it was not present.

In the model, low-pass-filtered activation acts as a subthreshold memory trace that increases the probability that sensory input induces detection of the probe stimulus. This is documented by the simulations for Experiment 2a (Figure 4B), in which the probability of detecting the probe at varied temporal gaps from the presentation of the object is compared to the baseline condition, in which no object stimulus is presented to the model.

The effect of the interstimulus interval on the persistent facilitation of probe visibility was not statistically significant in Experiment 2. However, as yet unpublished results, using the same adjustment method as in Experiment 10, have indicated that the measured visibility threshold was gradually reduced over the same time span as in Experiment 2. The reduction was too small to have impacted visibility judgments in Experiment 2 but likely would do so over longer interstimulus intervals.

Experiment 3 provided psychophysical evidence that the subthreshold activation persisting after a low-contrast object is no longer present can facilitate the visibility of a nearby probe. In the model, the memory trace (subthreshold activation) that is the residue of excitatory facilitation is spatially spread around the location of the object. This is crucial to understanding how visibility is stabilized when an object is moved to another location. The simulations in Figure 5 account for the observations of Experiment 3, in which the probe is shifted 8 pixels center-to-center (corresponding to 3.3 arcmin in the experiments) from the location at which the object was presented earlier, with a 614-ms temporal interval during which there is no stimulus input to the model. Compared with the baseline condition, detection probability at different levels of probe contrast was enhanced by the earlier presentation of the object.

Both Experiment 1 and 3 (Figures 3 and 5) exhibit a limitation of the model: At very low contrasts, the probability of probe visibility in the experiments does not seem to fall to zero, but the predicted probability does. In the model, the probability of visibility goes to zero for sufficiently weak contrast as there is no mechanism to generate detections for small enough input. In Experiments 5 to 9 (Figures 8 to 12), we do not see the same discrepancy between model and data. The somewhat more elevated levels of the probability of visibility at the lowest contrasts in Experiment 1 and 3 are not explained by the model.

### Bistable detection: Random fluctuations

In Experiments 1 and 3 there was a relatively wide range of contrasts over which perception was bistable; that is, at each of its contrast values, the probe was either visible or invisible, with the proportion of visibility increasing with contrast. A line segment (the object in this experiment) with contrast in the bistable range was the basis for Experiment 4, which assessed the stabilization of visibility over multiple unpredictable relocations. The object was visible during its first presentation for 62% of the trials and invisible for the other 38% of the trials. Visible/invisible bistability depended on whether or not activation-boosting self-excitation was elicited, which was determined by random fluctuations in detector activation.

In the model, stochasticity comes from neural noise that induces fluctuations in the no-stimulus resting level of the detector. The fluctuations evolve on a slow time scale, possibly reflecting trial-to-trial fluctuations in the observers’ perceptual sensitivity that is equivalent to a slowly varying background level of activation. When input is first presented, detection occurs when the background level of activation happens to be relatively high (i.e., when fluctuations raise the resting level), leading to visibility of the stimulus. Detection does not occur when the background level of activation happens to be relatively low (i.e., when fluctuations lower the resting level), leading to invisibility of the stimulus (see Figure 1 for an illustration). The simulations (Figure 7B) closely followed the experimental results. That is, the probability of detection on an initial trial was 0.62 and the probability of non-detection was 0.38. The fluctuating resting level may induce stochastic switches toward and away from detection, but the rate of these switches is relatively low in either direction, reflecting the fact that both detection and the non-detection levels of activation reflect stable states of neural activation.

### Bistable detection: Hysteresis

Further evidence for different activation levels associated with visibility and invisibility was obtained in a series of experiments assessing hysteresis. Using the modified method of limits, the same level of end-of-trial contrast was reached by either a sequence of gradually decreasing object contrast (descending trials, which began with a contrast level sufficient for visibility) or a sequence of gradually increasing object contrast (ascending trials, which began with a contrast too low for visibility). If “extra” activation elicited by self-excitation were not contributing to visibility for the descending trials, visibility would have been equally likely for descending and ascending trials with the same end-of-trial contrast. Contrary to this possibility, hysteresis was obtained in in five different experiments. That is, visibility at corresponding end-of-trial contrast levels was greater for the descending trials (with a preceding history of above-threshold activation) compared with the ascending trials (with a preceding history of below-threshold activation).

The results of simulation included in Figure 8 document how hysteresis emerges from the bistable neural dynamics of the model. Input strength varied from levels at which only the subthreshold activation state is stable, through the bistable regime, to the regime in which only the above-threshold activation state is stable. On ascending trials, activation typically remains in the subthreshold state until it becomes unstable in the detection instability (i.e., until self-excitation is engaged and activation exceeds the visibility threshold), whereas on descending trials activation typically remains in the self-excited, above-threshold activation state until it becomes unstable at the reverse detection instability (until activation falls below the visibility threshold). Neural noise may induce switches before reaching those limits, leading to the graded change of the probability of detection in both directions.

### Bistable detection without competition

Most past studies of perceptual bistability have involved competing perceptual states, each capable of above-threshold activation (e.g., the two orientations of the Necker cube) (Leopold, Wilke, Maier, & Logothetis, 2002) or two different motion directions for the motion quartet (Hock, Schoner, & Giese, 2003). The bistability of visibility and invisibility for the objects (line segments) studied here does not result from competition between neurons representing different perceptual outcomes. It results, instead, from different outcomes for a single neural representation: activation stabilized above threshold by self-excitation versus activation that remains below threshold when random fluctuations prevent activation from engaging self-excitation. Bistability between a perceptual state and its absence has been observed previously for binocular fusion (Fender & Julesz, 1967) and the integration of local motions to form a global motion pattern (Williams, Phillips, & Sekuler, 1986). Experiment 6 provided direct evidence for this asymmetry in the neural nature of the bistable perceptual states. Adaptation was enhanced by repeatedly presenting the *start*-*of-trial* contrast levels for ascending and descending trials. This pre-adaptation reduced hysteresis by reducing detector activation at the start of descending trials, thereby increasing the likelihood of visible-to-invisible switches. There was no corresponding increase in invisible-to-visible switches for ascending trials because there was no above-threshold activation to adapt. The simulations shown in Figure 9, alongside the experimental results, confirm this theoretical account for the asymmetry of the bistable perceptual states. Further confirmation came from the elimination adaptation from the model. It eliminated the simulated difference between trials with and without start-of-trial repetitions.

### Bistable detection: Switching without adaptation

Other than adaptation, stochastic switching to the alternate percept also has the potential to reduce hysteresis. To probe the role of stochastic switching, Experiment 7 provided additional opportunity for stochastic switching by repeatedly presenting the end-of-trial contrast. It was found that hysteresis was reduced for ascending trials because of switching from invisibility to visibility that occurred during these repetitions; that is, there was more switching with than without end-of-trial repetitions. The end-of-trial repetitions increased the opportunity for stochastic fluctuations to result in the engagement of activation-boosting excitatory interactions. Even if there had been adaptation to the below-threshold activation of ascending trials (as observed for motion quartets by Hock, Schöner, & Hochstein, 1996), the adaptation would have weakened activation and thus decreased the probability of invisible-to-visible switches. To our knowledge, the increased invisible-to-visible switches constituted the first direct psychophysical evidence for switching that was the sole result of random fluctuations in activation. This evidence for noise-induced switching in the absence of adaptation challenges numerous reports, going back to the “satiation” theory of Köhler and Wallach (1944), where switching for bistable stimuli depends on adaptation weakening the current percept while at the same time the alternative percept is recovering from adaptation (see also Ditzinger & Haken, 1989; Long, Toppino, & Mondin, 1992).

The simulations shown in Figure 10, alongside the experimental results from Experiment 7, confirm this theoretical account. Additional modeling results confirmed that these switches were the result of random fluctuations rather than adaptation. That is, invisible-to-visible switches within the end-of-trial repetitions continued to be simulated when adaptation was removed from the model.

### The memory trace and its spatial spread

The results of Experiments 2 and 3 indicate that activation induced by a previously visible object could enhance detection of a stimulus even after a blank interval of up to at least 800 ms duration. The theoretical account of the model attributes this facilitation to the memory trace of prior activation that leads to residual subthreshold activation in the vicinity of the activated location. Such preactivation at predictable locations is consistent with neurophysiological evidence that parietal neurons are preactivated in anticipation of a saccadic eye movement that would bring a visual stimulus to a predictable future location (Duhamel, Colby, & Goldberg, 1992). Experiment 8 showed, however, that such preactivation can occur at *un*predictable future locations. Hysteresis was obtained in Experiment 8 despite the insertion of 614-ms blank intervals between successive relocations of the object, intervals during which initially suprathreshold detector activation decays below the visibility threshold. Hysteresis also was obtained in the model simulations shown alongside the experimental results in Figure 11. In both the experiment and model, hysteresis is due entirely to the spread of preactivation from the memory trace to retinal locations surrounding the object.

It is possible, however, that the pre-shaping observed in Experiment 8 was limited to detectors in the center of the fovea. This was possible because frame durations of 360 ms were sufficiently long for observers to re-fixate on the object after successive re-presentations of the object. This possibility was ruled out by the results of Experiment 9. In this experiment, observers could not re-fixate following the relocation of the object; the average distance between successive relocations was doubled, and the duration at each location was reduced to 116 ms. Hysteresis was nonetheless obtained, indicating that the successive presentations of the object were linked by the spread of subthreshold activation to a retinal region surrounding the current location of the stimulus. It was not limited to detectors in the fovea. The simulations shown alongside the experimental results in Figure 12 were obtained by merely reducing the presentation duration of sensory input. The hysteresis obtained was again largely due to the memory trace, which builds across multiple presentations of the object. Experiment 10 (Figure 15) provided evidence that the spatial spread of excitation by low-contrast objects could provide the basis for the build-up of memory traces that result in the persistence of subthreshold activation in surrounding retinal locations.

Perceptual persistence across blank intervals has been observed for bistable stimuli with two competing perceptual states, each capable of above-threshold activation. These experiments (e.g., Orbach, Ehrlich, & Heath, 1963; Leopold et al., 2002) indicate that a previously established percept is restored when the bistable stimulus reappears, often after many minutes in which no stimulus is presented. These results have been attributed to biases formed by memories of preceding intervals during which one or the other percept dominates (Pastukov & Braun, 2008; Pastukov, García-Rodríguez, Haenicke, Guillamon, Deco, & Braun, 2013). The latter authors support this interpretation with a mathematical model that invokes different time courses for the build-up versus the decay of perceptual biases. These time courses are equivalent to the solutions of the dynamics of the memory trace used in our model (see Appendix B). Essentially, the neural field model can be viewed as a neural dynamic implementation of Pastukov and colleagues’ earlier account. A similar theoretical model has accounted for sequential effects in the perception of repeatedly presented ambiguous stimuli (with long blank intervals separating the repetitions). The model invokes both adaptation and memory (Noest, van Ee, Nijs, & van Wezel, 2007). Even though their mathematical formulation differs from ours, the two models are equivalent, with ours closer to the generic framework of neural dynamics (e.g., Gerstner et al., 2014).

### Collinear Gabor patterns

The empirical results obtained in the current study with laterally displaced, vertical line segments were, in many cases, consistent with previous evidence obtained in experiments with Gabor patterns—spatial Gaussians superimposed on sine gratings (Zenger & Sagi, 1996; Tanaka & Sagi, 1998). Target Gabor patterns were flanked, above and below by orientationally aligned (i.e., collinear) Gabor patterns. In Experiment 1 of the current study, the visibility of line segments (“probes”) was affected by nearby line segments (“objects”); probe visibility was facilitated by low-contrast objects and suppressed by high-contrast objects. Similarly, Zenger and Sagi (1996) found that the visibility of target Gabor patterns was facilitated by flanking, collinear Gabor patterns that were low in contrast and suppressed by flanking, collinear Gabor patterns that were high in contrast In Experiment 2, the visibility of a very low-contrast probe was facilitated by the earlier (up to 800 ms) presentation of a low-contrast object at the same location. Tanaka and Sagi (1998) similarly reported the facilitation of visibility for a Gabor pattern by a previously presented, low-contrast Gabor pattern. In their case, the facilitation bridged temporal gaps of up to 16 seconds.

There were, however, a number of findings in the current paradigm that were either inconsistent with or have not yet been addressed by experiments using collinear Gabor patterns. In their initial study with collinear gratings, Polat and Sagi (1993) found that the visibility of a target Gabor pattern is facilitated for relatively small target/flanker distances and suppressed for larger flanker/target distances. In Experiment 10, as well as in as yet unpublished experiments, there has been no indication that probe visibility is facilitated at short object/probe distances and suppressed at longer object/probe distances. Also, Tanaka and Sagi's (1998) evidence for the persistent effects of previously presented Gabor patterns, although consistent with the results of current Experiment 2, did not address whether memory traces of previous presentations could facilitate visibility at another, nearby location (as in Experiment 3) or at different, randomly determined locations (as in Experiment 9). The latter experiment provided evidence that the visibility of a low-contrast object at one location pre-shapes its visibility at its next location via persisting subthreshold activation of detectors surrounding the low-contrast object. Comparable research with Gabor patterns has not been done.

Experiments 1 to 3 of the current study provided the foundation for six subsequent experiments that investigated the stabilization of visibility over sequences of randomly determined relocations. These experiments, which entailed measurements of hysteresis and stochastic switching, point to a dynamical model that accounts for the evolution of detector activation as the contrast of an object increases or decreases over time. The dynamic neural field model, which simulated the results of the nine experiments reported in the current article, could conceivably account for the results obtained with collinear Gabor patterns. In fact, the model proposed by Adini et al. (1997) is mathematically very similar, postulating the same kind of excitatory and inhibitory populations that are coupled as in the present model. This early work, however, did not address the dynamics of activation and thus could not account for the hysteresis and stochastic switching effects observed in Experiments 4 to 9.

Additional results obtained with collinear Gabor patterns include effects on lateral interactions of attention (Freeman, Sagi, & Driver, 2001; Freeman, Sagi, & Driver, 2004), extended experience (Polat & Sagi, 1994), eccentric presentations (Shani & Sagi, 2005), and the organization of global configurations (Solomon & Morgan, 2000). These effects are potentially addressable within the framework of the current article, with emphasis on the distinction between low and high contrasts. A key question is whether there are detectors that respond selectively to low contrasts and are thus the basis for the determination of visibility thresholds, as well as independent detectors that respond selectively to higher contrast levels and are thus the basis for suprathreshold contrast matching (as in Georgeson & Sullivan, 1975), effects of attention (as in Freeman et al., 2001; Freeman et al., 2004), in contour integration (as in Williams & Hess, 1998), and in the formation of coherent objects (as in Solomon & Morgan, 2000).

## Conclusions

The experimental research and computational modeling reported in this article show that the visibility of low-contrast objects is stabilized over sequences of unpredictable relocations. The continued visibility is supported by self-excitation, which boosts activation, ensuring that it does not unstably hover near the visibility threshold. Continued visibility also is supported by short-term memory, which “bridges” intervals during which an object does not stimulate the retina. Because the relocations of objects were unpredictable in this study, the linkage between successive relocations, which was directly demonstrated by hysteresis effects, was mediated by the regional spread of subthreshold activation. Visibility is, of course, not a problem for high-contrast objects, so the extent to which self-excitation and short-term memory contribute to the linkage between successive relocations of high-contrast objects remains to be determined. The inhibitory effect of high-contrast objects is an important difference from the facilitatory effect of the low-contrast objects that were emphasized in the current article. Forthcoming research has uncovered other differences between the processing of low-contrast and high-contrast objects. Dynamic neural modeling is central to both the currently reported and forthcoming experiment research. It provides a unifying framework for understanding the interactive contributions of self-excitation, memory, adaptation, and inhibition to visual perception.

## Figures and Tables

**Figure 16.**
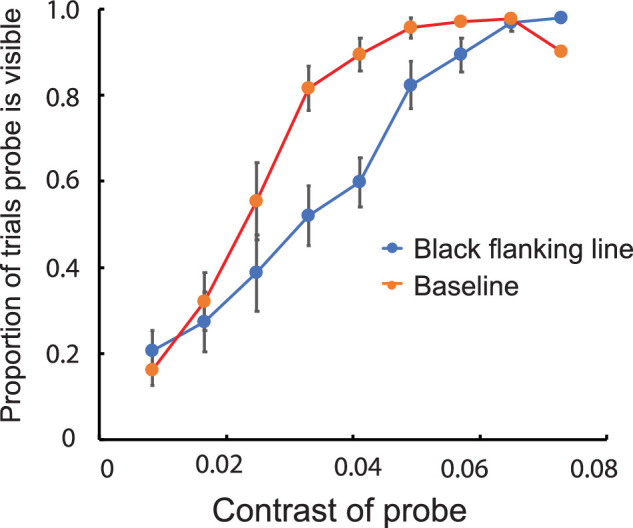
Results of preliminary experiment showing the effect of the black flanking line on the visibility of a nearby, variable-contrast white line. (In the baseline condition, the black flanking line was replaced by a small black dot, the marker.) The vertical line segments denote ±1 *SEM*.

## Appendix A

A preliminary experiment examined the effect of the 3.3 × 33.0-arcmin flanking black line on the visibility of a nearby white line of the same size. The nine contrast values of the white line were those tested in Experiments 2 to 9. The white line was resented to the right of either the black flanking line or a 3.2 × 3.2-arcmin black marker (the baseline condition). The gap between them was 3.3 arcmin. The black line (or black marker) and the white line were presented simultaneously at a different randomly determined location during every one-frame trial (duration = 360 ms).

The results indicated that the effect of the black flanker was inhibitory, reducing the visibility of the probe (Figure 16). The black flanker was presented alongside the probe during all of the experiments reported in this article in order to ensure the observer’s attention to the location of the white line when its contrast was very low. Its presence approximately linearizes the relationship between contrast and visibility over the range of contrasts studied in these experiments.

## Appendix B

At the core of the neural model (Figure 13) are an excitatory neural field, *u*(*x*, *t*), and an associated inhibitory neural field, *v*(*x*, *t*), both defined over visual space, *x*. The fields evolve in time, *t*, according to the neural dynamics:

$$\begin{array}{ccc} & & \tau_{u}\dot{u}\left(x,t\right)=-u\left(x,t\right)+h_{u}+c_{s}\left(x,t\right)stimulus\left(x,t\right)\\ & & \;+\;c_{selfexc}excitation\left(x,t\right)-c_{inh}inhibition\left(x,t\right)\\ & & \;+\;c_{m}memorytrace\left(x,t\right)+noise\\ & & \tau_{v}\dot{v}\left(x,t\right)=-v\left(x,t\right)+h_{v}+c_{exc}excitation\left(x,t\right) \end{array}$$

that may be viewed as mean-field approximations of the layered cortical neural networks (Amari, 1977). Here, the − *u*(*x*, *t*) and − *v*(*x*, *t*) terms are ultimately inherited from the dynamics of neural membranes and generate stable states to which activation converges on the times scales, τ_*u*_ = 15 ms and τ_*v*_ = 5 ms. In the absence of stimulus input, the resting levels, *h_u_* = −5 and *h_v_* = −8 are stable states: *u*(*x*, *t*) = *h_u_* and *v*(*x*, *t*) = *h_v_*.

The visual stimulus provides external input into the neural field, *u*(*x*, *t*). For example, a line segment of a given contrast at location *x_s_* provides input,

$$\begin{array}{c} Stimulus\left(x,t\right)=\frac{Contrast}{\sqrt{2\pi }\sigma_{s}}\exp\left[-\frac{{\left(x-x_{s}\right)}^{2}}{2\sigma_{s}^{2}}\right] \end{array}$$

where the Gaussian of width, σ_*s*_ = 2, models the effective forward connectivity from the retina to the neural field. Contrast is expressed in units of activation and ranges from 3.8 to 4.6 in the various simulations.

The output of either field is given by a sigmoidal threshold function,

$$\begin{array}{c} \sigma \left(z\right)=\frac{1}{1+\exp\left(-\beta z\right)} \end{array}$$

$z\in \left\{u,v\right\},\beta =5$ that emulates the threshold property of neural spike generation.

Recurrent connectivity is assumed to be homogeneous within the fields as described by Gaussian kernels:

$$\begin{array}{c} w_{k}\left(x-x^{'}\right)=\frac{1}{\sqrt{2\pi }\sigma_{k}}exp\left[-\frac{{\left(x-x^{'}\right)}^{2}}{2\sigma_{k}^{2}}\right] \end{array}$$

where *k* ∈ {*e*, *i*} with σ_*e*_ = 6 and σ_*i*_ = 8. Self-excitation is thus generated by input of strength, *c_selfexc_* = 7.3, to the neural field, *u*(*x*, *t*), that depends on the output, σ(*u*(*x*, *t*)), of that same field:

$$\begin{array}{c} Excitation\left(x,t\right)=\int dx^{'}w\left(x-x^{'}\right)\sigma \left(u\left(x^{'},t\right)\right) \end{array}$$

That same projection also provides excitatory input of strength, *c_exc_* = 12, to the inhibitory field, *v*(*x*, *t*). The inhibitory field projects onto and inhibits the neural field, *u*(*x*, *t*), through:

$$\begin{array}{c} Inhibition\left(x,t\right)=\int dx^{'}w\left(x-x^{'}\right)\sigma \left(v\left(x^{'},t\right)\right) \end{array}$$

with coupling strength *c_inh_* = 8.

The memory trace models a facilitory mechanism that evolves on the time scale, τ_*m*_ = 150 ms, according to

$$\begin{array}{c} \tau_{m}{\dot{u}}_{m}\left(x,t\right)=-u_{m}\left(x,t\right)+\sigma \left(u\left(x,t\right)\right). \end{array}$$

It could be viewed as a low-pass filter of suprathreshold activation, σ(*u*(*x*, *t*)). The memory trace provides input,

$$\begin{array}{c} Memorytrace\left(x,t\right)=\int dx^{'}w_{e}\left(x-x^{'}\right)u_{m}\left(x^{'},t\right), \end{array}$$

to the excitatory neural field that effectively increases the local resting level of the field. When and where activation, *u*(*x*, *t*), falls below zero, the memory trace decays on the slower time scale of 4000 ms.

Neural noise, critical to trial to trial variability and stochastic switching, is modeled as an Ornstein–Uhlenbeck process:

$$\begin{array}{c} \tau_{n}\frac{d}{dt}noise\left(t\right)=-noise\left(t\right)+q\xi \left(t\right) \end{array}$$

where ξ(*t*) is Gaussian white noise of unit variance, q=120 ms  is the standard deviation of the noise source, and τ_*n*_ = 8000 ms is the correlation time of the noise process. This provides, in effect, a fluctuating resting level that can be thought of as a slowly varying background activation level (a fluctuating resting level).

Adaptation is modeled as a weakening of sensory input according to the Hebbian rule:

$$\begin{array}{ccc} \tau_{a}{\dot{c}}_{s}\left(x,t\right) & = & \left(-c_{s}\left(x,t\right)+c_{min}\right)\sigma \left(u\left(x,t\right)\right)\\ & & \sigma \left(stimulus\left(x,t\right)\right)+\left(-c_{s}\left(x,t\right)+1\right)\\ & & \left[1-\sigma \left(u\left(x,t\right)\right)\sigma \left(stimulus\left(x,t\right)\right)\right] \end{array}$$

where τ_*a*_ = 1000 ms. Input strength is thus reduced from 1 to the minimal input strength *c_min_* = 0.85, when and where positive input induces positive activation. In the absence of positive input or positive activation, input strength recovers toward the default value of 1.

The spatial scale of the neural fields is defined in units of half a pixel where 1 pixel corresponds to 1.65 arcmin.

In numerical simulation, the Euler–Maruyama method was used to integrate the neural dynamics with a step size of 0.5 ms, spatially sampling the fields by 200 grid points. The stimulus conditions modeled each experiment as described in the main text. Psychophysical observations were emulated by making a detection decision on a given stimulus presentation when activation was above zero for at least 2/3 of a 7.5-ms time interval at the end of stimulus presentation. Reported means are based on 100 runs in each condition, seeding the random number generation only once. The values of model parameters were adjusted by hand to achieve fit of all nine experiments, constrained by a theoretical analysis of the different dynamic regimes in which the neural fields must operate to generate detection decisions only in the presence of significant stimulus input.

The parameter values listed in the table below were constrained by neurophysiology and the qualitative dynamics of the model. We briefly sketch these constraints here.

### Time scales

The time scales of activation, τ_*u*_ = 15 ms and τ_*v*_ = 4 ms, are aligned with the time scale of neural membrane dynamics, which are on the order of 10 ms (Gerstner et al., 2014). Inhibition, τ_*v*_, must be faster than excitation, τ_*u*_, to avoid oscillations. The time scale of the memory trace, τ_*m*_ = 150 ms, must be larger than the times scales of activation to fulfill its dynamic role as activation memory. It must be small enough for a single stimulus presentation to leave a trace (Experiments 2 and 3). We chose τ_*m*_ = 150 ms, one order of magnitude larger than τ_*u*_. The time scale of memory decays was chosen large enough to have no effect during a single presentation period, effectively neglecting decay. The time scale of adaptation, τ_*a*_, must be large enough for its effect to extend through the stimulus series of the hysteresis paradigm, on the time scale of seconds. We chose τ_*a*_ = 1000 ms. The time scale of noise,  τ_*n*_, was constrained by Experiment 4. The low switching rate observed there together with approximately equal initial probability of visibility and non-visibility suggests temporal correlations of activation fluctuations that extend throughout the duration of a trial, on the time scale of seconds. We chose τ_*n*_ = 8000 ms, although the precise values did not matter.

### Levels of activation

Given that the threshold of the sigmoid function is set to zero, the resting levels, *h_u_* and *h_v_*, must be negative, but are arbitrary otherwise. Together with the levels of input, *contrast*, they fix the units of activation. *h_v_* was chosen lower than *h_u_* to ensure that inhibitory activation reached threshold at larger inputs than excitatory activation, consistent with the observations of Experiment 1. (This could also have been achieved by weaker strength of coupling from the excitatory to the inhibitory layer, see below). The steepness of the sigmoid, β, determines the range of activation levels that the neural dynamics is sensitive to. At β = 5, the sigmoid covered a large portion of its range from 0 to 1 within the range of activation levels fixed by resting and input levels.

### Widths

The width of the Gaussian filter for input, σ_*s*_ = 2, was given the smallest possible value given the discretization of the fields in the numerical implementation (spatial step size =1). Its function is merely to avoid spatial discontinuities in input that may introduce numerical artifacts. The mapping from visual units to the field dimension was arbitrary, fixed to provide sufficient spatial resolution to sample the dependency of interaction on the spatial distance between object and probe studied in additional experimental work not included in this paper. Choice of the widths of the excitatory (σ_*e*_ = 6) and inhibitory (σ_*i*_ = 8) interaction kernel was informed by that empirical data. Inhibitory coupling must be broader than excitatory coupling to ensure stability.

### Coupling strengths

The three coupling strengths between the two layers of activation, *c_selfexc_* = 7.3, *c_exc_* = 27, and *c_inh_* = 8, were the actual fitting parameters of the model. Their adjustment was guided by the quality of the fits. These parameter values are constrained by the requirement that the fields are in a subthreshold state for sufficiently low levels of input, in a suprathreshold state for *u*, in a subthreshold state for *v* at intermediate levels of input, and in a suprathreshold state for both layers at sufficiently high levels of input (Experiment 1).

### Adaptation strength

The parameter *c_min_* = 0.85 determines the maximal amount by which effective input strength is lowered through adaptation (15%). Its value was constrained by Experiment 6.

### Noise level

The parameter q=120 ms  is constrained by the rate of switching in Experiment 4. Given the numerical effort to run stochastic simulations, we focused on tuning the coupling strengths, accepting rough estimates for all other parameters.

**Table:**

τ*_u_* = 15 ms
τ*_v_* = 4 ms
τ*_m_* = 150 ms (4000 ms for decay)
τ*_a_* = 1000 ms
τ*_n_* = 8000 ms
*h_u_* = –5
*h_v_* = –8
Contrast = 3.8 to 4.6
β = 5
σ*_s_* = 2
σ*_e_* = 6
σ*_i_* = 8
*c_selfexc_* = 7.3
*c_exc_* = 27
*c_inh_* = 8
*c_min_* = 0.85
$q=120\sqrt{ms}$
